# Application of spectral CT in coil embolization treatment of bronchial artery aneurysm: A case report

**DOI:** 10.34172/jcvtr.026.33417

**Published:** 2025-12-20

**Authors:** Shanshan Zhang, Tao Hu, Langzhou Fu, Longyu Duan

**Affiliations:** Department of Radiology, The Third Affiliated Hospital of Chongqing Medical University, Chongqing, China

**Keywords:** Bronchial artery anaurysm, Bronchial artery embolization, Spectral CT

## Abstract

We report a case of a bronchial artery aneurysm accompanied by a bronchial artery to pulmonary artery fistula in which only coil embolization was performed. Post-embolization, the majority of patients face significant metal artifacts from stents or coils during routine CT scans, which can compromise the visibility of the images. In this case, we conducted a spectral CT scan with metal artifact reduction technology postoperatively to clearly visualize the coils and accurately evaluate the treatment’s effectiveness.

## Introduction

 Bronchial artery aneurysm (BAA) is a rare and potentially life-threatening condition characterized by a high risk of rupture, which can result in severe hemoptysis. Studies have shown that the rupture of BAA is not related to the size of the aneurysm, hence immediate treatment is recommended upon detection of BAA.^1^

 The rapid evolution of CT technology has led to a broader adoption of spectral CT, especially in addressing the challenge of artifacts from metal implants in CT scans. The application of metal artifact reduction (MAR) technology in spectral CT has been extensively documented in the literature.^2^ In this case, we use the technique which effectively reduces the interference of metal-induced artifacts on the target structures and adjacent tissues, significantly improving the clarity and diagnostic value of the images.

## Case Presentation

 A 53-year-old female patient was admitted to the hospital with a one-month history of cough and hemoptysis accompanied by palpitations for four days. The patient had a dry cough for one month, and four days prior to admission, she began coughing up blood-tinged sputum, occasionally fresh blood, along with palpitations and discomfort, but without chest tightness or pain. She had a history of mechanical mitral valve replacement. Due to the patient’s main symptoms of hemoptysis and palpitations, a bronchial artery CT scan was planned.

**Figure 1:**
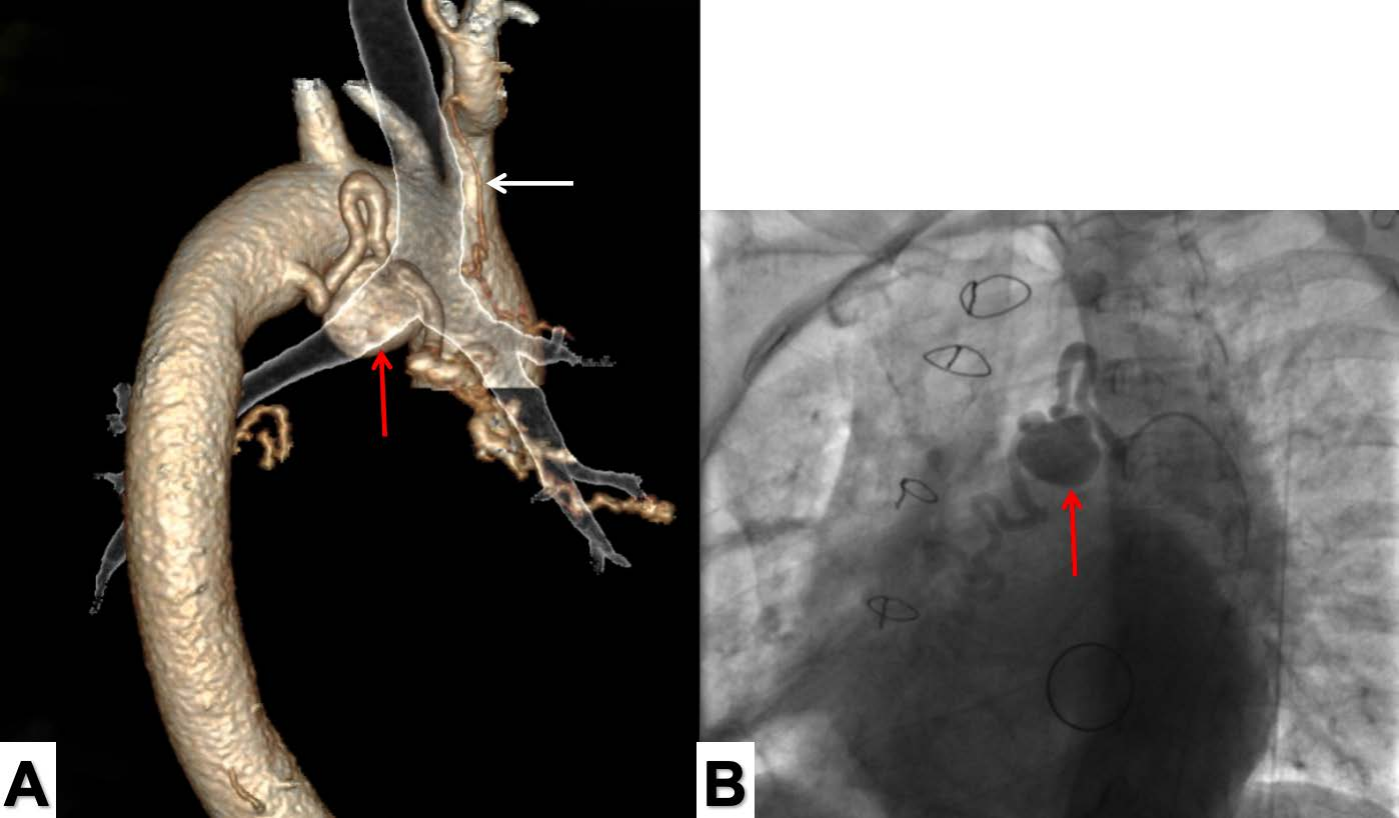
(A) The 3D vascular and trachea fusion image shows the bronchial artery aneurysm (red arrow) in relation to the bronchial position. The right ectopic bronchial artery (white arrow) emanates from the right subclavian artery. (B) Digital subtraction angiography showed the right bronchial artery aneurysm about 2.5x2.5cm in size (arrow) with a bronchial artery-pulmonary artery fistula

 The bronchial artery CT diagnosis indicated a R2L1 distribution of bronchial arteries, with the right bronchial artery originating from the lesser curvature of the aortic arch, with a tortuous course and local aneurysm formation; another ectopic bronchial artery on the right side originated from the right subclavian artery (Figure 1A). A bronchial artery embolization procedure was planned.

**Figure 2:**
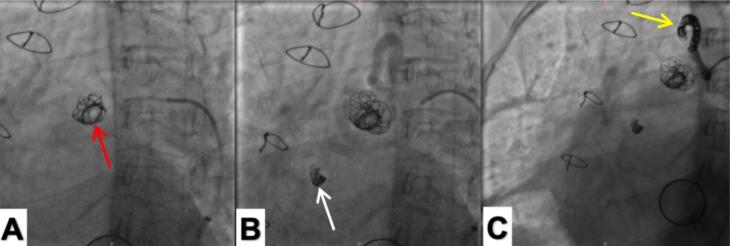
(A) The lumen of the bronchial artery aneurysm was embolized with five spring coils (red arrow). (B) The distal bronchial artery (bronchial artery-pulmonary artery fistula) was embolized with a spring coil to block the retrograde flow (white arrow). (C) The proximal end of the bronchial artery was embolized with spring coils to completely block the blood supply of the bronchial artery aneurysm (yellow arrow). Reexamination of the angiography showed that there was no development of the inflow end, lumen, and outflow end of the tumor, which proved that the embolization was complete

 The patient underwent a successful anesthesia, right femoral artery puncture was performed and 6F sheath catheter was placed. We found the bronchial artery opening on the small curved side of aortic arch, with aneurysmal dilation extending and one outflow artery running to the pulmonary artery, Partial pulmonary artery visualization. In combination with the preoperative bronchial artery CT, a BAA with a bronchial artery-pulmonary artery fistula was considered. After exchanging the vascular sheath for support and changing to a C2 catheter, the bronchial artery was selectively entered. Angiography indicated a BAA measuring approximately 2.5x2.5cm (Figure 1B), with contrast medium flowing into the lower bronchial artery of the right lung. After several attempts with a guidewire and micro catheter, the bronchial artery aneurysm was successfully accessed, and embolization was performed with five coils, the outflow and inflow ends of the aneurysm were embolized with six additional coils (Figure 2). A follow-up angiogram showed no contrast filling in the main bronchial artery, the aneurysm, and the outflow end, confirming complete embolization (Supplementary File 1, Video 1).

**Figure 3:**
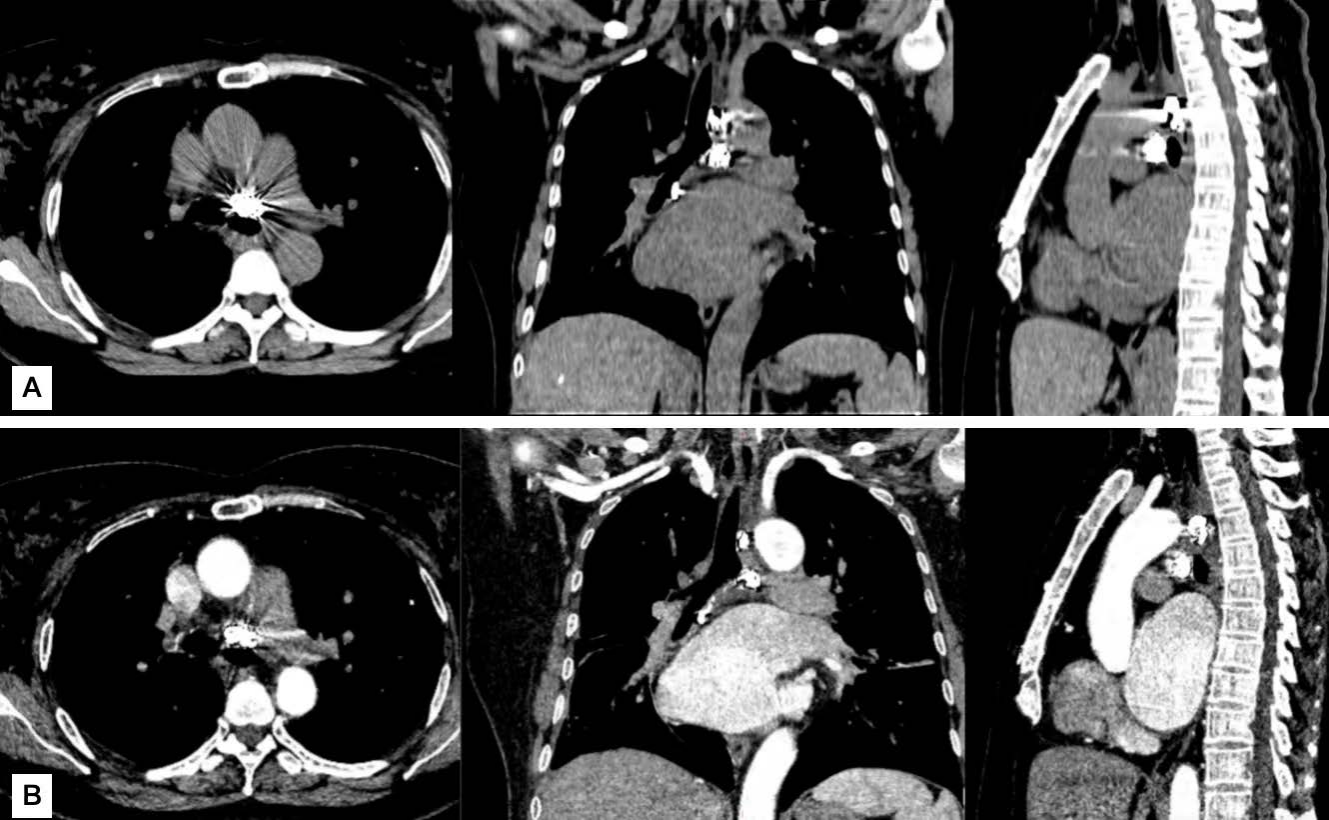
(A) Postoperative conventional CT scans in axial, coronal, and sagittal planes demonstrate significant metal artifacts from the coils, with surrounding tissue structures obscured by these artifacts and indistinguishable. Spectral MAR technique CT scans in axial, coronal, and sagittal planes show markedly reduced metal artifacts, allowing clear visualization of the coils and their surrounding tissue structures

**Figure 4:**
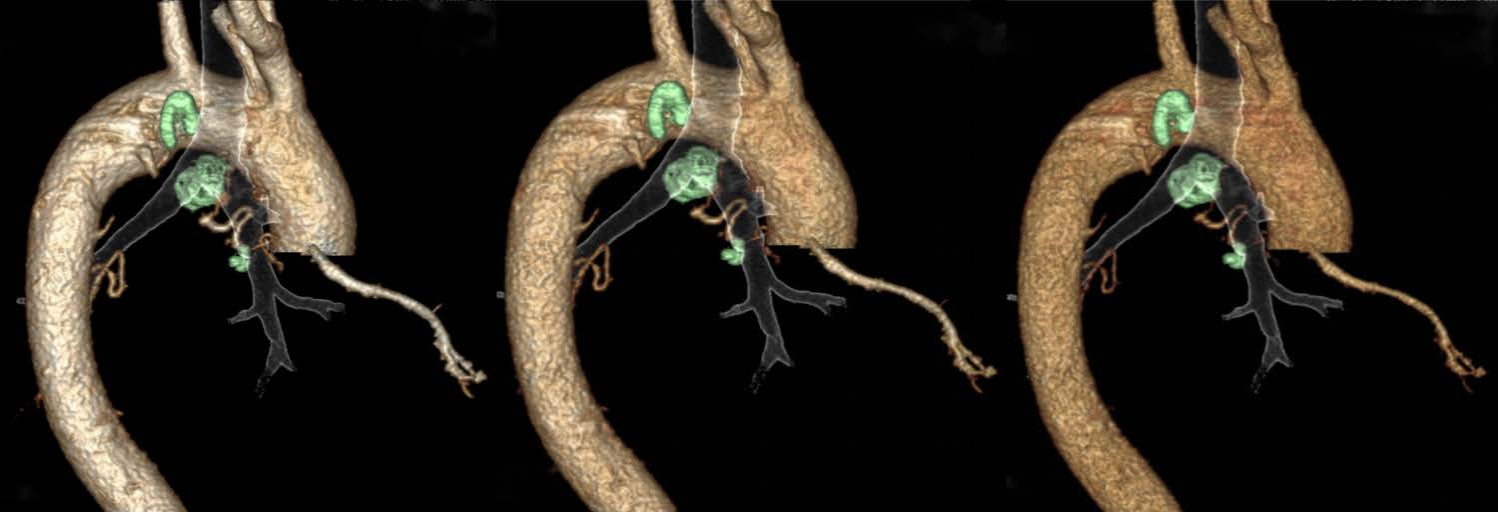
The three monoenergetic images (50 keV, 60 keV, and 70 keV from left to right) from spectral CT scanning clearly display the position and morphology of the bronchial artery and coils through vascular Volume Rendering

 A follow-up CT scan was performed five days postoperatively. Due to the effectiveness of the MAR technology in spectral CT, A comparison was made between the conventional CT scan and the spectral CT scan with MAR technology of the axial (Supplementary File 2, Video 2), coronal, and sagittal images at the level of the coils (Figure 3). The image comparison showed that the spectral CT with MAR technology effectively reduced the impact of metal artifacts from the coils on the surrounding tissues and vascular details in conventional scans, and clearly displayed the coil morphology and the status of BAA embolization. The spatial relationship between the bronchial artery coils, the aorta, and the bronchus is more intuitively demonstrated through Volume Rendering images (Figure 4).

## Discussion

 BAA are very rare but life-threatening diseases. The existing body of research on BAA is limited, and it is typically classified based on anatomical location into mediastinal, intrapulmonary, or a combination of both. The exact cause of BAA is not well understood, and the clinical manifestations vary, depending on the location of the aneurysm and whether it has ruptured, since rupture can be life-threatening, BAA should be treated immediately after diagnosis, and endovascular coil embolization has become the preferred treatment method for BAA.^3^

 For bronchial artery aneurysms (BAA) with a short vascular segment between the aneurysm and the aorta, combined treatment with trans catheter arterial embolization and aortic covered stent placement is the preferred approach, as it effectively isolates the feeding artery of the aneurysm.^4-9^ However, some studies have reported risks such as air embolism and increased spinal ischemia associated with covered stent placement, leading to the use of controlled coil embolization alone.^10^ Matsumoto et al used a simple aortic stent to completely isolate blood flow into the aneurysm by securely sealing the opening of the feeding artery.^11^ In this case of BAA, the treatment plan involved only coil embolization. Due to the large size of the BAA and the sufficiently long vascular segment between the aneurysm and the aorta, filling the aneurysm cavity with five coils resulted in insignificant reduction in aneurysm size, with contrast agent still flowing through. Additionally, due to the presence of a bronchial artery-pulmonary artery fistula and the patient’s financial constraints, complete filling of the aneurysm cavity was not pursued. Instead, coil embolization was performed at the outflow end of the bronchial artery to block retrograde blood flow, while coil embolization at the opening end of the aneurysm aimed to completely block blood flow to the BAA. These individual cases demonstrate that the choice of treatment varies depending on the aneurysm neck and the degree of arterial tortuosity. However, there is no definitive reporting on how to evaluate the specific length of the aneurysm neck, as BAA itself is not highly prevalent, and each reported case differs. Therefore, the optimal treatment plan must be selected based on the specific characteristics of the BAA.

 An increasing number of metal implants, such as stents, artificial joints, and coils, are being used in clinical diagnosis and treatment. These implants often cause severe metal artifacts on conventional CT scans, making it difficult to distinguish the surrounding tissue structures and complicating post-treatment efficacy evaluation. The rapid development of spectral CT has addressed this challenge. The GE Revolution CT we used, equipped with spectral metal artifact reduction (MAR) technology, can eliminate most radial artifacts from metal implants, clearly displaying the morphological features of the implants and the surrounding tissue structures.^12^ According to existing literature, whether treated with coils alone or in combination with aortic stent placement, these cases exhibited significant metal hardening artifacts on follow-up CT scans, obscuring the relationship between the coils and their surrounding structures.^4-10^ in our case, the use of spectral CT with metal artifact reduction technology allowed clear visualization of the positional relationship between the bronchial artery aneurysm coils and the surrounding tissues and vessels. Additionally, we applied spectral CT’s optimized vascular imaging technology, selecting different monoenergetic images to reconstruct three-dimensional fusion images of the coils within the bronchial artery, the blood vessels, and the bronchi. This provided a more intuitive observation of the positional and morphological characteristics of the coils. Of course, long-term case follow-up and further experience with similar cases are needed.

## Conclusion

 BAA are rare but pose significant risks. Immediate treatment is essential upon detection. The choice of therapeutic approach should be tailored based on the morphology, location, size, and rupture status of the BAA. For postoperative regular follow-ups, Spectral CT combined with MAR technology can be utilized for evaluating treatment efficacy.

## Competing Interests

 The authors declare no conflict of interest.

## Ethical Approval

 An institutional ethical approval was obtained for this work from The Third Affiliated Hospital of Chongqing Medical University Sciences and a signed written informed consent form was obtained from patient. (Ethical Code: 2023-KL-074).

## Supplementary Files

Supplementary File 1, Video 1. The Complete Procedure Video of Bronchial Artery Aneurysm Embolization with Interventional Coils.

Supplementary File 2, Video 2. Comparison of Conventional and Spectral CT Scans in Axial Views Following Bronchial Artery Aneurysm Coil Embolization.
